# *catena*-Poly[[[aqua­(pyridine-4-carboxyl­ato-κ*N*)silver(I)]-μ-hexa­methyl­ene­tetra­amine-κ^2^ *N*:*N*′] dihydrate]

**DOI:** 10.1107/S1600536810050452

**Published:** 2010-12-08

**Authors:** Dajun Sun, Liying Han, Hu Zang

**Affiliations:** a Department of Vascular Surgery, The China–Japan Union Hospital of Jilin University, Changchun 130033, People’s Republic of China; b Department of Gynecology, The Second Hospital of Jilin University, Changchun 130041, People’s Republic of China; c Department of Orthopedics, The China–Japan Union Hospital of Jilin University, Changchun 130033, People’s Republic of China

## Abstract

In the title compound, {[Ag(C_6_H_4_NO_2_)(C_6_H_12_N_4_)(H_2_O)]·2H_2_O}_*n*_, the Ag^I^ atom shows a distorted triangular pyramidal geometry,, formed by two N atoms from two hexa­methyl­ene­tetra­amine (hmt) ligands and one N atom from a pyridine-4-carboxyl­ate (4-pdc) ligand and one water mol­ecule. The hmt ligands bridge the Ag atoms, forming a chain along [001]. The carboxyl­ate group of the 4-pdc ligand is uncoordinated. O—H⋯O hydrogen bonds between the water mol­ecules and carboxyl­ate groups stabilize the structure.

## Related literature

For general background to the design and synthesis of coordination polymers, see: Eddaoudi *et al.* (2001 ▶).
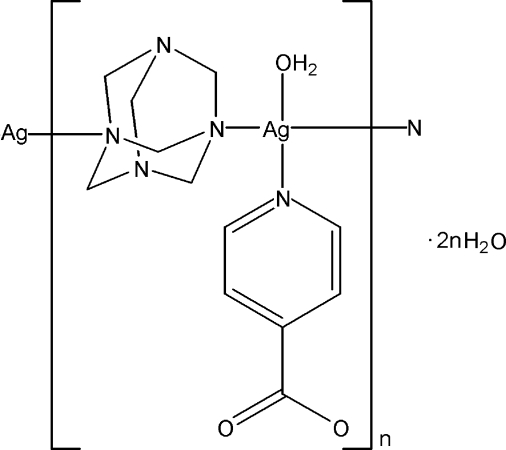


## Experimental

### 

#### Crystal data

[Ag(C_6_H_4_NO_2_)(C_6_H_12_N_4_)(H_2_O)]·2H_2_O
*M* *_r_* = 424.22
Orthorhombic, 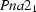

*a* = 11.8271 (5) Å
*b* = 13.2122 (5) Å
*c* = 10.2560 (4) Å
*V* = 1602.62 (11) Å^3^
*Z* = 4
Mo *K*α radiation
μ = 1.29 mm^−1^
*T* = 293 K
0.24 × 0.20 × 0.19 mm

#### Data collection

Bruker APEX CCD diffractometer
Absorption correction: multi-scan (*SADABS*; Sheldrick, 1996 ▶) *T*_min_ = 0.747, *T*_max_ = 0.792
7849 measured reflections
2380 independent reflections
2347 reflections with *I* > 2σ(*I*)
*R*_int_ = 0.016

#### Refinement

*R*[*F* ^2^ > 2σ(*F* ^2^)] = 0.016
*wR*(*F* ^2^) = 0.041
*S* = 1.08
2380 reflections
226 parameters
10 restraints
H atoms treated by a mixture of independent and constrained refinement
Δρ_max_ = 0.20 e Å^−3^
Δρ_min_ = −0.55 e Å^−3^
Absolute structure: Flack (1983 ▶), 876 Friedel pairs
Flack parameter: 0.01 (2)

### 

Data collection: *SMART* (Bruker, 2007 ▶); cell refinement: *SAINT* (Bruker, 2007 ▶); data reduction: *SAINT*; program(s) used to solve structure: *SHELXS97* (Sheldrick, 2008 ▶); program(s) used to refine structure: *SHELXL97* (Sheldrick, 2008 ▶); molecular graphics: *SHELXTL* (Sheldrick, 2008 ▶) and *DIAMOND* (Brandenburg, 1999 ▶); software used to prepare material for publication: *SHELXTL*.

## Supplementary Material

Crystal structure: contains datablocks global, I. DOI: 10.1107/S1600536810050452/hy2384sup1.cif

Structure factors: contains datablocks I. DOI: 10.1107/S1600536810050452/hy2384Isup2.hkl

Additional supplementary materials:  crystallographic information; 3D view; checkCIF report

## Figures and Tables

**Table 1:** Selected bond lengths (Å)

Ag1—N1	2.287 (2)
Ag1—N2	2.256 (2)
Ag1—N5^i^	2.306 (2)
Ag1—O1*W*	2.673 (2)

Symmetry code: (i) 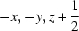.

**Table 2:** Hydrogen-bond geometry (Å, °)

*D*—H⋯*A*	*D*—H	H⋯*A*	*D*⋯*A*	*D*—H⋯*A*
O1*W*—H1*A*⋯O2*W*^ii^	0.85 (3)	1.91 (3)	2.743 (3)	169 (3)
O1*W*—H1*B*⋯O1^iii^	0.85 (1)	1.91 (2)	2.714 (2)	157 (3)
O2*W*—H2*A*⋯O2	0.84 (1)	1.88 (1)	2.722 (3)	173 (3)
O2*W*—H2*B*⋯O3*W*^iv^	0.84 (3)	1.99 (3)	2.787 (3)	161 (3)
O3*W*—H3*A*⋯O1*W*^v^	0.85 (1)	1.95 (1)	2.788 (3)	169 (3)
O3*W*—H3*B*⋯O1	0.85 (1)	1.89 (1)	2.728 (2)	169 (3)

Symmetry codes: (ii) 
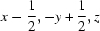
; (iii) 
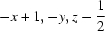
; (iv) 
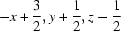
; (v) 
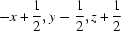
.

**Fig. 1.**
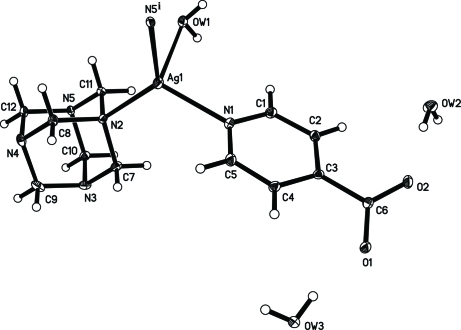
The asymmetric unit of the title compound. Displacement ellipsoids are drawn at the 30% probability level. [Symmetry code: (i) -x, -y, z+1/2].

**Fig. 2.**
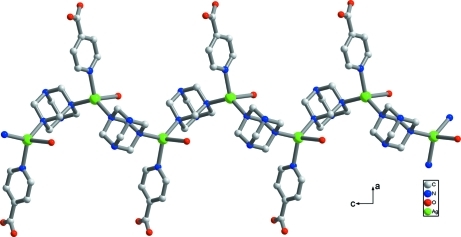
View of the chain structure in the title compound.

## supplementary crystallographic information

### Comment

The design and synthesis of coordination polymers have been a research field of rapid expansion not only because of their fascinating structures, but also owing to their interesting properties as new functional materials of tremendous potential applications in molecular recognition, ion-exchange, and catalysis for reactions (Eddaoudi *et al.*, 2001). In this work, the reaction of pyridine-4-carboxylatic acid (4-Hpdc) and hexamethylenetetraamine (hmt) with Ag^I^ ion yielded a new coordination polymer.

As shown in Fig. 1, the asymmetric unit of the title compound contains one Ag^I^ atom, one 4-pdc ligand, one hmt ligand, one coordinated water molecule and two uncoordinated water molecules. The Ag1 atom shows a distorted triangle pyramidal geometry, completed by three N atoms from two hmt ligands and one 4-pdc ligand and one O atom from a water molecule. The hmt ligands bridge the Ag atoms, forming a one-dimensional chain. The carboxylate group of the 4-pdc ligand is uncoordinated. O—H···O hydrogen bonds between the water molecules and carboxylate groups stabilize the structure.

### Experimental

A mixture of 4-Hpdc (0.615 g, 0.5 mmol), Ag(NO_3_)_2_ (0.085 g, 0.5 mmol) and hmt (0.070 g, 0.5 mmol) in water was heated at 150°C in a Teflon-lined stainless steel autoclave for 5 d. The reaction system was then slowly cooled to room temperature. Crystals suitable for X-ray diffraction analysis were collected by filtration.

### Refinement

C-bound H atoms were positioned geometrically and refined using a riding mode, with C—H = 0.93 and 0.97 Å and *U*_iso_(H) = 1.2*U*_eq_(C). The water H atoms were located in a difference Fourier map and refined with restraints of O—H = 0.85 (1) and H···H = 1.38 (1) Å and *U*_iso_(H) = 1.5*U*_eq_(O).

### Crystal data

**Table:**

[Ag(C_6_H_4_NO_2_)(C_6_H_12_N_4_)(H_2_O)]·2H_2_O	*F*(000) = 864
*M**_r_* = 424.22	*D*_x_ = 1.758 Mg m^−^^3^
Orthorhombic, *P**n**a*2_1_	Mo *K*α radiation, λ = 0.71073 Å
Hall symbol: P 2c -2n	Cell parameters from 2380 reflections
*a* = 11.8271 (5) Å	θ = 3.0–26.1°
*b* = 13.2122 (5) Å	µ = 1.29 mm^−^^1^
*c* = 10.2560 (4) Å	*T* = 293 K
*V* = 1602.62 (11) Å^3^	Block, colorless
*Z* = 4	0.24 × 0.20 × 0.19 mm

### Data collection

**Table:**

Bruker APEX CCD diffractometer	2380 independent reflections
Radiation source: fine-focus sealed tube	2347 reflections with *I* > 2σ(*I*)
graphite	*R*_int_ = 0.016
φ and ω scans	θ_max_ = 25.0°, θ_min_ = 2.3°
Absorption correction: multi-scan (*SADABS*; Sheldrick, 1996)	*h* = −13→14
*T*_min_ = 0.747, *T*_max_ = 0.792	*k* = −15→15
7849 measured reflections	*l* = −10→12

### Refinement

**Table:**

Refinement on *F*^2^	Secondary atom site location: difference Fourier map
Least-squares matrix: full	Hydrogen site location: inferred from neighbouring sites
*R*[*F*^2^ > 2σ(*F*^2^)] = 0.016	H atoms treated by a mixture of independent and constrained refinement
*wR*(*F*^2^) = 0.041	*w* = 1/[σ^2^(*F*_o_^2^) + (0.0231*P*)^2^ + 0.3127*P*] where *P* = (*F*_o_^2^ + 2*F*_c_^2^)/3
*S* = 1.08	(Δ/σ)_max_ < 0.001
2380 reflections	Δρ_max_ = 0.20 e Å^−^^3^
226 parameters	Δρ_min_ = −0.55 e Å^−^^3^
10 restraints	Absolute structure: Flack (1983), 876 Friedel pairs
Primary atom site location: structure-invariant direct methods	Flack parameter: 0.01 (2)

### Fractional atomic coordinates and isotropic or equivalent isotropic displacement parameters (Å^2^)

**Table:**

	*x*	*y*	*z*	*U*_iso_*/*U*_eq_	
Ag1	0.128363 (13)	0.013803 (11)	0.15278 (4)	0.02268 (7)	
C1	0.3975 (2)	0.04853 (19)	0.1327 (3)	0.0235 (6)	
H1	0.3756	0.0961	0.0708	0.028*	
C2	0.51160 (18)	0.03798 (16)	0.1607 (3)	0.0213 (5)	
H2	0.5645	0.0788	0.1190	0.026*	
C3	0.5467 (2)	−0.03375 (17)	0.2511 (2)	0.0180 (5)	
C4	0.4633 (2)	−0.09081 (18)	0.3108 (3)	0.0226 (5)	
H4	0.4826	−0.1395	0.3724	0.027*	
C5	0.3513 (2)	−0.0753 (2)	0.2786 (3)	0.0253 (6)	
H5	0.2968	−0.1145	0.3200	0.030*	
C6	0.6705 (2)	−0.04864 (19)	0.2834 (2)	0.0195 (5)	
C7	0.1034 (2)	−0.1856 (2)	−0.0106 (3)	0.0232 (6)	
H7A	0.1690	−0.1589	−0.0556	0.028*	
H7B	0.1299	−0.2228	0.0651	0.028*	
C8	−0.0697 (2)	−0.14419 (19)	0.0997 (2)	0.0214 (5)	
H8A	−0.0454	−0.1812	0.1764	0.026*	
H8B	−0.1189	−0.0898	0.1281	0.026*	
C9	−0.0568 (2)	−0.29341 (18)	−0.0278 (3)	0.0298 (6)	
H9A	−0.0977	−0.3395	−0.0842	0.036*	
H9B	−0.0321	−0.3312	0.0482	0.036*	
C10	0.0045 (2)	−0.19809 (17)	−0.2111 (2)	0.0216 (5)	
H10A	−0.0355	−0.2433	−0.2696	0.026*	
H10B	0.0696	−0.1715	−0.2573	0.026*	
C11	−0.0086 (2)	−0.04628 (17)	−0.0847 (3)	0.0188 (5)	
H11A	−0.0572	0.0093	−0.0586	0.023*	
H11B	0.0560	−0.0179	−0.1301	0.023*	
C12	−0.1700 (2)	−0.15591 (19)	−0.1010 (3)	0.0207 (5)	
H12A	−0.2195	−0.1012	−0.0741	0.025*	
H12B	−0.2126	−0.2005	−0.1579	0.025*	
N1	0.31739 (18)	−0.00721 (15)	0.1914 (2)	0.0226 (6)	
N2	0.03133 (16)	−0.10028 (15)	0.0333 (2)	0.0175 (4)	
N3	0.04299 (17)	−0.25501 (15)	−0.0976 (2)	0.0238 (4)	
N4	−0.13283 (16)	−0.21224 (17)	0.0138 (2)	0.0225 (5)	
N5	−0.07120 (15)	−0.11312 (15)	−0.1745 (2)	0.0165 (4)	
O1	0.69699 (14)	−0.13139 (13)	0.33372 (19)	0.0263 (4)	
O2	0.73760 (15)	0.02175 (13)	0.2588 (2)	0.0294 (5)	
O1W	0.11516 (14)	0.16451 (15)	−0.0205 (2)	0.0254 (4)	
H1A	0.145 (2)	0.2117 (19)	0.024 (3)	0.038*	
H1B	0.161 (2)	0.147 (2)	−0.080 (2)	0.038*	
O2W	0.73929 (16)	0.19124 (15)	0.1080 (2)	0.0344 (5)	
H2A	0.736 (2)	0.1415 (17)	0.159 (2)	0.052*	
H2B	0.777 (3)	0.176 (2)	0.042 (2)	0.052*	
O3W	0.60916 (15)	−0.31522 (14)	0.4000 (3)	0.0330 (5)	
H3A	0.5402 (12)	−0.313 (2)	0.423 (3)	0.049*	
H3B	0.629 (2)	−0.2569 (14)	0.373 (4)	0.049*	

### Atomic displacement parameters (Å^2^)

**Table:**

	*U*^11^	*U*^22^	*U*^33^	*U*^12^	*U*^13^	*U*^23^
Ag1	0.01850 (10)	0.02679 (10)	0.02274 (11)	−0.00070 (6)	−0.00291 (10)	−0.00824 (17)
C1	0.0231 (11)	0.0237 (11)	0.0236 (19)	0.0030 (9)	−0.0032 (12)	0.0047 (12)
C2	0.0200 (10)	0.0214 (10)	0.0226 (12)	−0.0030 (8)	0.0014 (14)	0.0042 (15)
C3	0.0193 (12)	0.0163 (11)	0.0185 (13)	0.0030 (9)	−0.0024 (10)	−0.0034 (10)
C4	0.0203 (12)	0.0220 (12)	0.0256 (14)	0.0024 (9)	0.0013 (11)	0.0062 (11)
C5	0.0201 (12)	0.0263 (14)	0.0295 (15)	−0.0025 (10)	0.0028 (11)	0.0048 (12)
C6	0.0177 (12)	0.0225 (12)	0.0184 (13)	0.0003 (10)	0.0004 (10)	0.0004 (10)
C7	0.0202 (12)	0.0234 (14)	0.0262 (16)	0.0054 (10)	−0.0018 (12)	−0.0043 (12)
C8	0.0206 (12)	0.0248 (13)	0.0188 (12)	−0.0023 (10)	0.0012 (10)	0.0021 (10)
C9	0.0414 (16)	0.0176 (13)	0.0305 (15)	−0.0053 (11)	−0.0100 (13)	0.0024 (11)
C10	0.0216 (12)	0.0223 (12)	0.0208 (13)	0.0025 (9)	−0.0006 (11)	−0.0039 (10)
C11	0.0179 (12)	0.0157 (10)	0.0228 (13)	−0.0002 (9)	0.0000 (10)	0.0016 (10)
C12	0.0154 (12)	0.0257 (13)	0.0208 (13)	−0.0046 (10)	−0.0008 (11)	0.0032 (11)
N1	0.0158 (10)	0.0237 (10)	0.0284 (16)	0.0010 (8)	−0.0010 (9)	−0.0004 (8)
N2	0.0132 (9)	0.0191 (10)	0.0202 (11)	0.0020 (8)	−0.0010 (8)	−0.0002 (8)
N3	0.0290 (11)	0.0179 (10)	0.0244 (11)	0.0055 (9)	−0.0075 (10)	−0.0041 (9)
N4	0.0232 (11)	0.0252 (12)	0.0190 (12)	−0.0077 (8)	−0.0014 (9)	0.0053 (9)
N5	0.0150 (10)	0.0167 (10)	0.0178 (11)	−0.0032 (8)	0.0000 (8)	0.0014 (8)
O1	0.0186 (8)	0.0235 (9)	0.0366 (11)	−0.0002 (7)	−0.0035 (8)	0.0104 (8)
O2	0.0190 (9)	0.0265 (10)	0.0427 (13)	−0.0057 (7)	−0.0055 (9)	0.0098 (8)
O1W	0.0196 (9)	0.0265 (11)	0.0300 (12)	−0.0025 (7)	0.0035 (8)	−0.0021 (9)
O2W	0.0317 (10)	0.0319 (10)	0.0396 (12)	0.0047 (8)	0.0038 (9)	0.0147 (8)
O3W	0.0219 (9)	0.0206 (10)	0.0564 (15)	−0.0012 (7)	−0.0013 (10)	0.0072 (10)

### Geometric parameters (Å, °)

**Table:**

Ag1—N1	2.287 (2)	C8—H8B	0.9700
Ag1—N2	2.256 (2)	C9—N4	1.463 (3)
Ag1—N5^i^	2.306 (2)	C9—N3	1.470 (4)
Ag1—O1W	2.673 (2)	C9—H9A	0.9700
C1—N1	1.342 (3)	C9—H9B	0.9700
C1—C2	1.387 (3)	C10—N3	1.459 (3)
C1—H1	0.9300	C10—N5	1.484 (3)
C2—C3	1.390 (4)	C10—H10A	0.9700
C2—H2	0.9300	C10—H10B	0.9700
C3—C4	1.384 (3)	C11—N5	1.476 (3)
C3—C6	1.513 (3)	C11—N2	1.482 (3)
C4—C5	1.380 (3)	C11—H11A	0.9700
C4—H4	0.9300	C11—H11B	0.9700
C5—N1	1.330 (3)	C12—N4	1.461 (3)
C5—H5	0.9300	C12—N5	1.501 (3)
C6—O2	1.249 (3)	C12—H12A	0.9700
C6—O1	1.249 (3)	C12—H12B	0.9700
C7—N3	1.465 (3)	O1W—H1A	0.85 (3)
C7—N2	1.483 (3)	O1W—H1B	0.85 (1)
C7—H7A	0.9700	O2W—H2A	0.84 (1)
C7—H7B	0.9700	O2W—H2B	0.84 (3)
C8—N4	1.464 (3)	O3W—H3A	0.85 (1)
C8—N2	1.492 (3)	O3W—H3B	0.85 (1)
C8—H8A	0.9700		
			
N2—Ag1—N1	120.67 (7)	N3—C10—N5	112.10 (19)
N2—Ag1—N5^i^	130.41 (7)	N3—C10—H10A	109.2
N1—Ag1—N5^i^	102.86 (7)	N5—C10—H10A	109.2
N2—Ag1—O1W	96.13 (7)	N3—C10—H10B	109.2
N1—Ag1—O1W	105.23 (6)	N5—C10—H10B	109.2
N5^i^—Ag1—O1W	94.00 (7)	H10A—C10—H10B	107.9
N1—C1—C2	122.6 (3)	N5—C11—N2	112.42 (18)
N1—C1—H1	118.7	N5—C11—H11A	109.1
C2—C1—H1	118.7	N2—C11—H11A	109.1
C1—C2—C3	119.8 (2)	N5—C11—H11B	109.1
C1—C2—H2	120.1	N2—C11—H11B	109.1
C3—C2—H2	120.1	H11A—C11—H11B	107.9
C4—C3—C2	116.9 (2)	N4—C12—N5	111.27 (19)
C4—C3—C6	121.5 (2)	N4—C12—H12A	109.4
C2—C3—C6	121.6 (2)	N5—C12—H12A	109.4
C5—C4—C3	119.8 (2)	N4—C12—H12B	109.4
C5—C4—H4	120.1	N5—C12—H12B	109.4
C3—C4—H4	120.1	H12A—C12—H12B	108.0
N1—C5—C4	123.4 (2)	C5—N1—C1	117.3 (2)
N1—C5—H5	118.3	C5—N1—Ag1	119.57 (17)
C4—C5—H5	118.3	C1—N1—Ag1	123.05 (17)
O2—C6—O1	125.1 (2)	C11—N2—C7	107.5 (2)
O2—C6—C3	118.3 (2)	C11—N2—C8	107.76 (18)
O1—C6—C3	116.6 (2)	C7—N2—C8	107.63 (19)
N3—C7—N2	112.35 (19)	C11—N2—Ag1	106.48 (14)
N3—C7—H7A	109.1	C7—N2—Ag1	112.35 (14)
N2—C7—H7A	109.1	C8—N2—Ag1	114.77 (15)
N3—C7—H7B	109.1	C10—N3—C7	108.4 (2)
N2—C7—H7B	109.1	C10—N3—C9	108.41 (19)
H7A—C7—H7B	107.9	C7—N3—C9	108.1 (2)
N4—C8—N2	111.90 (19)	C12—N4—C9	108.8 (2)
N4—C8—H8A	109.2	C12—N4—C8	109.0 (2)
N2—C8—H8A	109.2	C9—N4—C8	108.17 (19)
N4—C8—H8B	109.2	C11—N5—C10	107.94 (17)
N2—C8—H8B	109.2	C11—N5—C12	107.64 (19)
H8A—C8—H8B	107.9	C10—N5—C12	108.15 (19)
N4—C9—N3	112.48 (19)	C11—N5—Ag1^ii^	106.63 (14)
N4—C9—H9A	109.1	C10—N5—Ag1^ii^	114.39 (15)
N3—C9—H9A	109.1	C12—N5—Ag1^ii^	111.83 (14)
N4—C9—H9B	109.1	H1A—O1W—H1B	108.9 (15)
N3—C9—H9B	109.1	H2A—O2W—H2B	110.2 (16)
H9A—C9—H9B	107.8	H3A—O3W—H3B	108.7 (15)

Symmetry codes: (i) −*x*, −*y*, *z*+1/2; (ii) −*x*, −*y*, *z*−1/2.

### Hydrogen-bond geometry (Å, °)

**Table:**

*D*—H···*A*	*D*—H	H···*A*	*D*···*A*	*D*—H···*A*
O1W—H1A···O2W^iii^	0.85 (3)	1.91 (3)	2.743 (3)	169 (3)
O1W—H1B···O1^iv^	0.85 (1)	1.91 (2)	2.714 (2)	157 (3)
O2W—H2A···O2	0.84 (1)	1.88 (1)	2.722 (3)	173 (3)
O2W—H2B···O3W^v^	0.84 (3)	1.99 (3)	2.787 (3)	161 (3)
O3W—H3A···O1W^vi^	0.85 (1)	1.95 (1)	2.788 (3)	169 (3)
O3W—H3B···O1	0.85 (1)	1.89 (1)	2.728 (2)	169 (3)

Symmetry codes: (iii) *x*−1/2, −*y*+1/2, *z*; (iv) −*x*+1, −*y*, *z*−1/2; (v) −*x*+3/2, *y*+1/2, *z*−1/2; (vi) −*x*+1/2, *y*−1/2, *z*+1/2.
